# Shed GP of Ebola Virus Triggers Immune Activation and Increased Vascular Permeability

**DOI:** 10.1371/journal.ppat.1004509

**Published:** 2014-11-20

**Authors:** Beatriz Escudero-Pérez, Valentina A. Volchkova, Olga Dolnik, Philip Lawrence, Viktor E. Volchkov

**Affiliations:** Molecular Basis of Viral Pathogenicity, CIRI, INSERM U1111- CNRS UMR5308, Université de Lyon, Université Claude Bernard Lyon 1, Ecole Normale Supérieure de Lyon, Lyon, France; Division of Clinical Research, United States of America

## Abstract

During Ebola virus (EBOV) infection a significant amount of surface glycoprotein GP is shed from infected cells in a soluble form due to cleavage by cellular metalloprotease TACE. Shed GP and non-structural secreted glycoprotein sGP, both expressed from the same GP gene, have been detected in the blood of human patients and experimentally infected animals. In this study we demonstrate that shed GP could play a particular role during EBOV infection. In effect it binds and activates non-infected dendritic cells and macrophages inducing the secretion of pro- and anti-inflammatory cytokines (TNFα, IL1β, IL6, IL8, IL12p40, and IL1-RA, IL10). Activation of these cells by shed GP correlates with the increase in surface expression of co-stimulatory molecules CD40, CD80, CD83 and CD86. Contrary to shed GP, secreted sGP activates neither DC nor macrophages while it could bind DCs. In this study, we show that shed GP activity is likely mediated through cellular toll-like receptor 4 (TLR4) and is dependent on GP glycosylation. Treatment of cells with anti-TLR4 antibody completely abolishes shed GP-induced activation of cells. We also demonstrate that shed GP activity is negated upon addition of mannose-binding sera lectin MBL, a molecule known to interact with sugar arrays present on the surface of different microorganisms. Furthermore, we highlight the ability of shed GP to affect endothelial cell function both directly and indirectly, demonstrating the interplay between shed GP, systemic cytokine release and increased vascular permeability. In conclusion, shed GP released from virus-infected cells could activate non-infected DCs and macrophages causing the massive release of pro- and anti-inflammatory cytokines and effect vascular permeability. These activities could be at the heart of the excessive and dysregulated inflammatory host reactions to infection and thus contribute to high virus pathogenicity.

## Author Summary

Ebola virus, a member of the Filoviridae family, causes lethal hemorrhagic fever in man and primates, displaying up to 90% mortality rates. Viral infection is typified by an excessive systemic inflammatory response resembling septic shock. It also damages endothelial cells and creates difficulty in coagulation, ultimately leading to haemorrhaging, organ failure and death. A unique feature of EBOV is that following infection high amounts of truncated surface GP, named shed GP, are released from infected cells and are detected in the blood of patients and experimentally infected animals. However the role of shed GP in virus replication and pathogenicity is not yet clearly defined. Here we show that shed GP released from virus-infected cells binds and activates non-infected DCs and macrophages causing the massive release of pro- and anti-inflammatory cytokines and also affects vascular permeability. These activities could be at the heart of the excessive and dysregulated inflammatory host reactions to infection and thus contribute to high virus pathogenicity.

## Introduction

Ebola virus (EBOV) is the causative agent of a haemorrhagic fever in humans responsible for sporadic outbreaks in several African countries including the current epidemic in Guinea, Liberia and Sierra Leone [1],[2] and for which there is currently no vaccine or approved treatment [3]. Fatal EBOV infection involves massive disseminated viral replication and host immune disregulation with uncontrolled cytokine secretion (for review, see [4]) and a disseminated intravascular coagulation syndrome resembling septic shock [5], [6].

EBOV is a member of the *Mononegavirales* order *Filoviridae* family. Its genome consists of seven genes and encodes seven structural and at least one non-structural protein [7]–[9]. Due to RNA editing phenomenon, transcription of the GP gene results in the synthesis of several GP gene specific mRNAs coding for viral glycoproteins including non-structural sGP and surface virion GP [10], [11]. Both glycoproteins are synthesized as a precursor molecule that is proteolytically cleaved by the cellular protease furin during intracellular processing [12]. sGP forms dimers [10], [13] whereas the cleaved carboxy-terminal fragment, termed delta peptide, is a monomer [14]. Viral surface spikes are formed as a trimer of GP_1,2_, made up of two subunits: GP_1_ and GP_2_ linked by a disulfide bond [15], [16]. GP_1_ is known to mediate virus attachment to host cells whereas GP_2_ is involved in membrane fusion [11], [17]. GP_1,2_ is a type I glycoprotein containing multiple N- and O-linked glycans [7], [18]. The majority of O-glycans are grouped in a region termed mucin-like domain [18]–[20].

A unique feature of EBOV is that following infection, virus encoded glycoproteins are released from cells in soluble forms. High amounts of sGP and truncated surface GP are detected in the blood of patients and experimentally infected animals [11], [21]. Release of sGP is explained by its secretion via the classical secretory pathway due to the lack of a transmembrane anchor [11], [22]. Cleavage of surface GP by the cellular metalloprotease TACE (TNFα-converting enzyme), a member of the ADAM (*a d*isintegrin *a*nd *metalloproteinase*) proteinase family, is responsible for GP shedding [21]. In this manner soluble shed GP resembles virion GP_1,2_, and has been shown to bind and sequester virus-neutralizing antibodies directed against surface GP [21]. Aside from this antibody-neutralizing activity the role of shed GP in virus replication and pathogenicity has not yet been clearly defined.

Following EBOV infection, primary virus replication occurs in dendritic cells (DCs) and macrophages and is then followed by massive replication in hepatocytes and splenocytes [23]–[26]. It has recently been revealed that monocytes are also involved in virus replication albeit in an entry-delayed and differentiation–dependent fashion [27]. Massive release of cytokines, chemokines and vasoactive substances follows the course of infection thus promoting the development of inflammatory disorders and playing an important role in EBOV pathogenesis [24]–[26]. Overall, excessive inflammatory responses to infection contribute to massive infiltration of virus target cells to sites of infection but also result in the observed septic shock-like syndrome. Remarkably, little cytokine secretion is detected following EBOV infection of DCs which can be explained by their impaired maturation upon infection [26], [28]–[30]. Monocytes also appear to secrete inflammatory cytokines when infected, but at lower levels [27]. Based on in vitro data macrophages are the only cells that produce cytokines at relatively high levels following EBOV infection [26], [28]. Using EBOV VLPs it has been demonstrated that cytokine and chemokine secretion from macrophages and DCs is likely to be dependent on GP [19], [30], [31] whereas the question remains as to how these host responses are triggered during infection.

In this study we identify the cellular targets for EBOV shed GP and answer the question if soluble EBOV glycoproteins could trigger the release of cytokines from non-infected immune cells. We show that shed GP but not sGP activates human-derived DCs and macrophages and induces the secretion of pro- and anti-inflammatory cytokines. We demonstrate that this activation can be diminished by anti-TLR4 antibodies or by addition of sera lectin MBL. Moreover, we show that the glycosylation pattern of shed GP is important for the activation of cytokine release. We also demonstrate the ability of shed GP to affect endothelial cell permeability both directly and indirectly through cytokine release. Our results suggest that shed GP could play an important role in viral pathogenicity.

## Results and Discussion

### Production and characterization of recombinant EBOV soluble glycoproteins

During EBOV infection significant amounts of soluble glycoproteins, including shed GP and secreted sGP, are released from virus-infected cells [21]. The role that these proteins might play in virus replication and pathogenicity has not yet been clearly defined. In effect, EBOV shed GP is structurally identical to virion surface GP except for the lack of its carboxy-terminal part consisting of 13 amino acids upstream of the membrane anchor, the anchor itself and a short cytoplasmic tail [21], [32]. Consequently, it was of interest to investigate whether shed GP and potentially sGP, that shares 295 amino-terminal amino acids with surface GP, can interact with and affect the function of DCs and macrophages that constitute an important and primary line of immune defense but that are also the major primary targets for viral infection in vivo. While transient expression of sGP from plasmid provides sufficient amounts of the protein secreted into the medium, the low efficiency of GP shedding seen with transient expression systems has hampered production of large enough quantities of this protein equivalent to those observed during EBOV infection. Of note, TACE, an enzyme responsible for GP shedding, is activated during EBOV infection [33] and the efficiency of shedding from virus-infected cells appears to be significantly higher. On the other hand the extent of glycosylation and post-translational modifications of EBOV GP prevents the use of more efficient expression systems such as bacteria or baculovirus. To surmount these difficulties we substituted two amino acids immediately surrounding the TACE cleavage site at positions D637A and Q638V and thus expected to increase efficiency of GP shedding as this newly generated sequence resembles that previously shown to be recognized by TACE for efficient release of TNFα [34]. The plasmid expressing this mutant was designated phCMVGP-HS and GP expressed from this plasmid as GP-HS (high shedding). Shed GP expressed by this mutant contains a single amino acid substitution at the carboxy-terminal end of shed GP. According to structural predictions it is highly unlikely that this single amino acid change would alter the structure or function of shed GP. Another difficulty faced in the production of recombinant GP of EBOV results from its strong cytotoxic effects [20], [35], [36] causing cell rounding, loss of cell attachment, and possibly the release of cellular factors due to eventual cell death. In order to address solely the potential functions of shed GP and to allow for possible effects caused by cytotoxicity, an EBOV GP mutant lacking shedding properties was used in this study as a negative control. This mutant contains a substitution L635V (GP-LS, low shedding) that was previously shown to prevent TACE cleavage and result in increased surface GP expression [21]. Of note, expression of this mutant is expected to result in similar, if not higher, cytotoxicity than that caused by wild-type GP (GP-WT) and would provide culture supernatants containing the same potential extracellular factors released from GP expressing cells whilst displaying undetectable amounts of shed GP.

**Figure 1:**
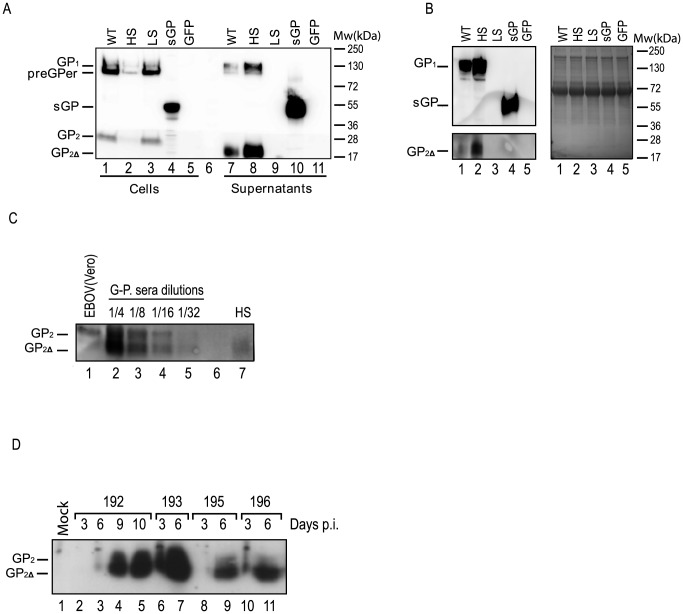
Production of recombinant EBOV shed GP and analysis of shed GP release in the blood of infected guinea pigs. (**A**) 293T cells were transfected with plasmids expressing GP-WT (WT), GP-HS (HS), GP-LS (LS), sGP and GFP and 36 h post-transfection cells and culture supernatants were collected and analyzed by Western Blot using anti-GP_1_ and anti-GP_2_ antibodies. GP_1_, endoplasmic reticulum precursor GP (preGPer), sGP, GP_2_, and truncated GP_2Δ_ are indicated. (**B**) Culture supernatants from 293 T cells expressing EBOV glycoproteins were concentrated using concentrator tubes (Pierce) and analyzed by Coomassie staining following SDS-PAGE (right panel). Samples were also analyzed by Western blot with appropriate antibodies (left panel). (**C**). Guinea pigs (strain Hartley) were infected intraperitoneally with 500 TCID50/animal of guinea pig-adapted EBOV [82]. Serial dilutions of sera collected at day 6 post-infection (5 µl, dilutions 1/4, 1/8, 1/16 & 1/32, lanes 2–5), supernatant from EBOV-infected Vero E6 cells (20 µl, EBOV Vero, lane 1) and a sample of concentrated recombinant shed GP (5 µl, HS, lane 6) were analyzed by Western blot using anti-GP2 antibodies. Estimation of shed GP amounts in the sera of animals in comparison with shed GP from GP-HS expressing cells was performed using ImageQuantTL software (GE Lifesciences). Sera of animals contained approximately 85 times higher amounts of shed GP than those used in experiments with immune cells. (**D**). Samples of sera collected at different intervals post infection from Mock (day 3 p.i.) - and EBOV-infected guinea pigs (animal numbers 192, 193, 195 & 196) as indicated, were analyzed by Western blot using anti-GP2 antibodies. The release of shed GP dramatically increased during the course of infection. Sera of infected animals contains both virion GP and shed GP as demonstrated by the presence of full-length virion-associated GP_2_ and truncated GP_2_ (GP_2Δ_).

In this manner, 293T cells were transfected with the plasmids expressing mutated GPs, GP-WT and sGP as well as GFP as a non-relevant control. Western blot analysis of cell lysates and culture supernatants revealed as expected, an important difference in efficacy of GP shedding between GP-WT and mutated GPs (Figure 1A). Of note, due to the removal of the region spanning the transmembrane anchor, after cleavage by TACE the GP_2_ within shed GP showed a lower molecular mass and faster migration on SDS-polyacrylamyde gels compared to GP_2_ in intracellular GP_1,2_ (compare lines 1 and 7; lines 2 and 8). While similar amounts of intracellular GP (represented by endoplasmic reticulum GP precursor - preGPer and mature GP_1,2_) were seen in cells expressing either GP-WT or GP-LS (compare lines 1 and 3), GP-LS did not show detectable levels of shed GP in the medium when compared with GP-WT. Notably, relatively low amounts of intracellular GP were detected with GP-HS whilst increased levels of shed GP were detected in the medium. As expected EBOV sGP is efficiently secreted from 293T cells transfected with corresponding plasmid (Figure 1A, lane 10). Using Vivaspin concentrators the volume of culture supernatants was reduced by 10 fold to increase the concentration of shed GP in supernatant samples. Of note, no significant differences were detected in total amount of proteins between samples (2 mg/ml), as also illustrated in Figure 1B by Coomassie staining. The presence of shed GP in concentrated samples from GP-HS expressing cells and its absence in samples from GP-LS expressing cells was confirmed by Western Blot analysis (Figure 1B, left panel, line 3).

Using serial dilutions of a commercially available recombinant baculovirus-expressed EBOV GP lacking the transmembrane domain we were able to estimate the amounts of shed GP and sGP in concentrated supernatants by western blot analysis and quantification software. The standard curve was linear over the range used for quantification and the GP-HS sample was found to contain ∼1 µg/ml and sGP ∼18 µg/ml (Figure S1 A and B). In subsequent experiments with immune cells shed GP (GP-HS) was typically used at a concentration of ∼0.4 µg/ml and sGP at a concentration of ∼7.2 µg/ml.

To avoid using the produced recombinant shed GP in subsequent experiments at concentrations superior to those observed with infection the quantity of shed GP in concentrated samples was compared with that present in the blood of experimentally infected animals. For this purpose groups of guinea pigs were inoculated intraperitoneally with 500 infectious units (TCID_50_) of guinea pig-adapted recombinant EBOV [37]. Infected guinea pigs were monitored for clinical manifestations and showed an increase in temperature starting by day 3 post-infection. When animals reached the ethical end point of infection they were euthanized. Blood was collected at different intervals post infection and the sera were assayed for the presence of shed GP in comparison with recombinant-produced shed GP. As demonstrated in Figure 1C and 1D the amounts of shed GP present in the blood of animals significantly exceed those used in our study. Quantification of shed GP in the sera based on amounts of truncated GP_2_ reveals approximately 85 times higher concentrations of shed GP (34 µg/ml) in infected animals than those used in this study. Furthermore the analysis of sera samples collected at different intervals post infection showed that the amounts of shed GP in the blood of animals significantly increased during the course of infection (Figure 1D), which is likely to be explained by both virus-induced activation of TACE [34] and also by spreading of infection that increases the number of virus infected cells.

Of note, our GP construction and production approach differed from that used in an earlier study investigating role of soluble EBOV glycoproteins [32]. In the previous study to address the role of shed GP the authors had used purified amino-terminal HA-tagged soluble glycoprotein produced through the addition of a stop codon immediately after the TACE cleavage site that allows efficient secretion of this truncated GP into the extracellular media. However, we have observed that when GP is produced through truncation of its transmembrane anchor it does not appear to wholly represent shed GP. Whilst truncated forms of GP had been previously used for crystal structure analysis of surface GP, and were principally shown to form trimers [18], the stability of such truncated oligomeric structures is suspected to be significantly lower in cell culture conditions. Indeed, as evidenced by our sedimentation analysis the transmembrane-anchor truncated GP mutant is mostly a monomer in solution (Figure S2). In contrast shed GP produced in our study is a trimer as was also observed during EBOV infection [21] and is in agreement with the trimeric structure of surface GP. Of note the process of shedding includes proper GP oligomerization, maturation, and transport to the cell surface where the mature GP spikes are cleaved by cellular TACE and shed from the cells.

In addition, in the previous study the soluble recombinant glycoproteins were produced in conditions containing human sera [32] which are known to contain sera lectins, including human MBL [38]–[40]. Indeed, MBL is a C-type lectin capable of acting as a pattern recognition molecule for pathogens by recognizing heavily glycosylated proteins, particularly those containing a high-mannose type of glycosylation such as that seen with EBOV surface GP [41]–[45]. Importantly, MBL has recently been shown capable of binding EBOV surface GP [42], [46], [47]. The presence of such sera lectins during production of soluble GP could hamper the investigation of the interaction of this protein with its cellular targets and therefore the expression and production of shed GP, sGP and also of all control proteins used in this study was performed in cells cultured in FCS free medium (VP-SFM, Gibco).

### EBOV soluble glycoproteins bind to DC and macrophages

**Figure 2:**
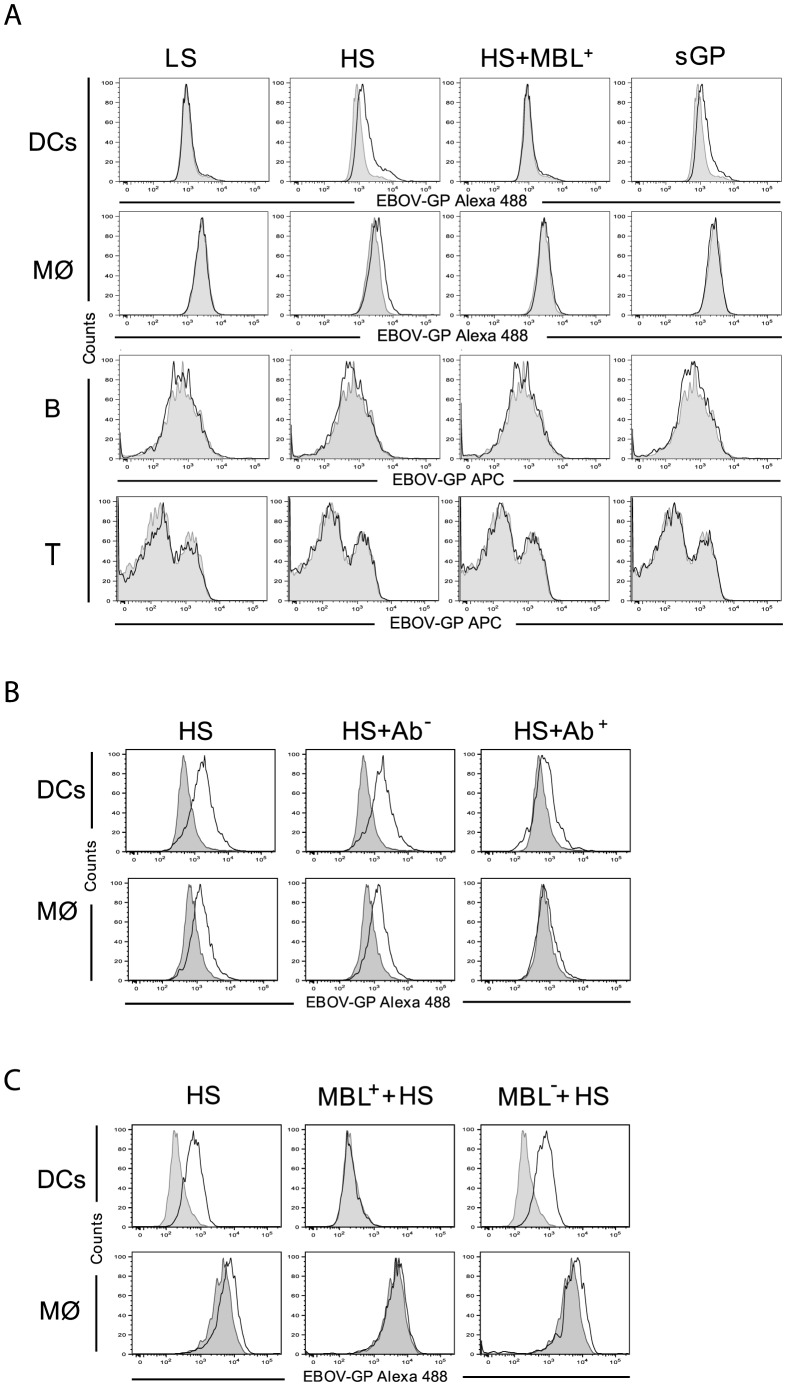
EBOV shed GP binding to DCs and macrophages. (**A**) Human monocyte-derived dendritic cells (DCs), monocyte-derived macrophages (MØ), and PBLs (shown B lymphocytes, B) were incubated with the concentrated culture supernatants described as well as shed GP in the presence of MBL-containing sera (150 ng/ml, HS+MBL^+^). Bound proteins were detected by subsequent incubation with mouse anti-GP1 antibodies and anti-mouse Alexa 488 coupled antibodies (DCs and MØ) and anti-mouse APC (B lymphocytes). Fraction of B lymphocytes was stained using CD20-FITC antibodies (Beckman Coulter). Shed GP binding to cells was analyzed by flow cytometry (**B**) DCs and MØ were either incubated with supernatants containing GP-HS (as above) or were pre-treated with anti-TLR4 antibody (Ab^+^) or isotypic control antibodies (Ab^−^) prior to shed GP treatment. Shed GP binding to cells was analyzed by flow cytometry. (**C**) DCs and MØ were incubated with serum containing 150 ng/ml of MBL-containing sera (MBL^+^), MBL-deficient sera (MBL^−^) or culture media alone before washing and incubation with shed GP (as above). Filled histograms represent staining with an isotype control antibody. Data shown are representative of three independent experiments with at least three different donors. For quantitative data and statistics see figure S3.

Next, human monocyte-derived DCs and macrophages (Mø) as well as peripheral blood lymphocytes (PBL) were incubated with the samples of culture medium containing the soluble EBOV GPs described above. To assess the ability of shed GP to bind to DCs, Mø or specific fractions of lymphocytes, flow cytometry was performed using anti-GP specific antibodies. DCs were shown to bind both shed GP and sGP while Mø could only bind shed GP (Figures 2A and S3A). As expected, incubation of the cells with GP-LS failed to evidence any GP specific binding in agreement with undetectable levels of shed GP in samples. It appears that neither B nor T lymphocytes (Figures 2A and S3A) bind shed GP or sGP in agreement with earlier data revealing an absence of surface GP binding to these cells [48], [49].

In light of data revealing an interaction between surface GP and MBL [42], [46], we also investigated whether MBL present in human sera is capable of affecting the binding of shed GP to cells. Of note, normal human plasma contains MBL concentrations ranging from 10 to 10000 ng/ml [50], [51]. Using commercially available human sera (Statens Serum Institute, Denmark) we demonstrate that pre-incubation of shed GP with such sera (final MBL concentration of 150 ng/ml) blocked shed GP binding to both DCs and Mø (Figures 2A and S3A).

Previously it was demonstrated that surface GP of EBOV interacts with toll-like receptor 4 (TLR4) [52], a cellular surface receptor that is known to recognize highly glycosylated molecules containing O-linked mannosyl residues such as those present on GP. It was thus of interest to investigate if shed GP could also mediate its activity through TLR4. To address this question DCs and Mø were pre-treated with mouse anti-TLR4 antibody (HTA125) and then incubated with shed GP. As a control, the cells were first incubated with mouse isotype control antibodies and then with shed GP. Binding of shed GP to both DCs and Mø was reduced upon treatment of the cells with anti-TLR4 antibody whereas no effect on GP binding was observed when control antibodies were used (Figures 2B and S3B). These results suggest that shed GP binding to DCs and Mø likelyinvolves its interaction with TLR4. In this regard an absence of shed GP binding to PBLs is in agreement with little to no surface TLR4 expression on lymphocytes from healthy donors [53]–[55].

It is worthy to note that MBL can also bind TLR4 [56]–[58]. In this regard the MBL-mediated reduction in shed GP binding to the cells could be explained either by the interference of MBL in shed GP binding to TLR4 and/or by sequestering shed GP via direct interaction with MBL. In an attempt to clarify this question we performed experiments where cells were pre-treated with sera containing MBL with subsequent washing and incubation with shed GP. As demonstrated in Figures 2C and S3C, shed GP binding to the cells was also blocked under these conditions, in this case most likely via interaction of TLR4 with MBL. In agreement, pre-incubation of cells with MBL deficient sera did not affect shed GP binding.

Thus far data obtained clearly indicate an interaction between shed GP and DCs and Mø. This is contrary to a previous publication in which no evidence of an interaction was found between shed GP and immune cells [32]. In this regard, the avoidance of sera lectins during GP production appears to be of vital importance as we demonstrate that the addition of human sera containing MBL abrogates binding of shed GP to target cells in vitro. Of note, as demonstrated in Figure 1C significantly higher amounts of shed GP are present in the blood of EBOV-infected guinea pigs than in our experimental conditions. Furthermore the continuous and massive release of shed GP during infection (Figure 1D) most likely would reduce or counteract the inhibitory effect of sera lectins in vivo even further.

Importantly, the data obtained in this study suggest that TLR4 is likely to be an important cellular partner involved in binding of shed GP to DCs and Mø. As TLR4 is abundantly expressed on macrophages and DCs [59]–[61] our findings led us to investigate further the role that such an interaction might play, in terms of activation of these cells and the induction of the release of pro-inflammatory cytokines that is known to be a characteristic of EBOV pathogenesis [62], [63].

### EBOV shed GP induces upregulation of cytokine transcription in both DCs and Mø

**Figure 3:**
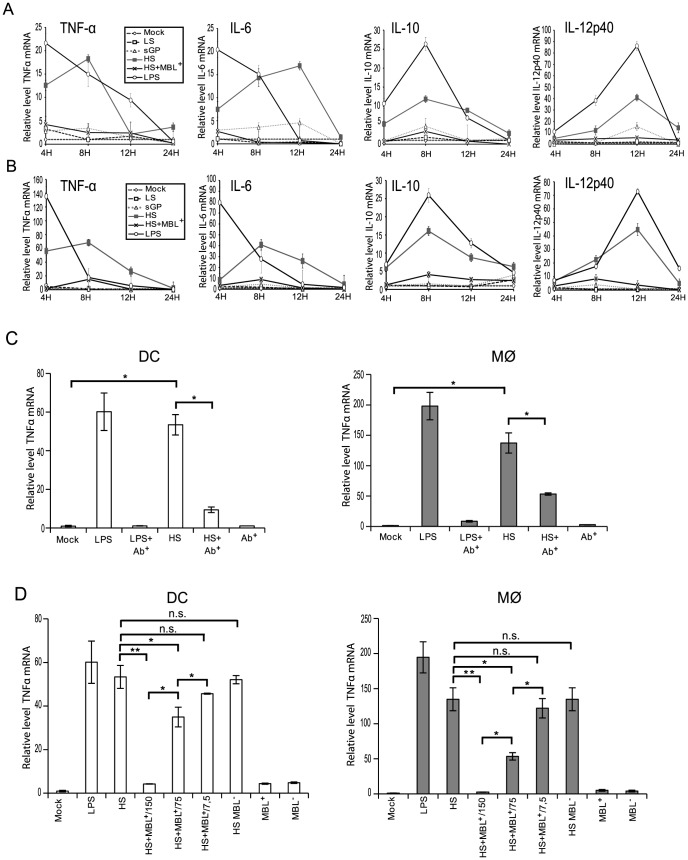
Shed GP induces transcriptional activation of cytokines in DCs and MØ. DCs and MØ (5×10^5^ cells) were incubated with concentrated culture supernatants as above (as Figure 2) and with LPS (500 pg/ml). Cells were collected at 4, 8, 12 and 24 h post-incubation as indicated. Expression levels of mRNA encoding TNF-α, IL-6, IL-10 IL-12p40, and GAPDH were measured by RT-Q-PCR in DCs (**A**) and MØ (**B**). Relative levels of the cytokines were obtained by normalization to GAPDH and Mock as indicated. (**C**) DCs and MØ as above were treated with concentrated culture supernatants for 8 h as follows: culture medium alone (Mock), 500 pg/ml LPS, shed GP (HS) or the cells were pre-incubated with anti-TLR4 antibody (Ab^+^) and then treated with shed GP or LPS. Expression levels of mRNAs encoding TNFα and GAPDH were analyzed by RT-Q-PCR as above. Relative levels of TNFα mRNA were obtained by normalization to GAPDH and Mock. (**D**) DCs and MØ were treated with concentrated culture supernatants as above. In addition HS culture supernatants were pre-incubated with different concentrations of MBL-containing sera as indicated (150 ng/ml, 75 ng/ml and 7.5 ng/ml) before addition to DCs or MØ. An MBL-deficient serum (MBL^−^) was used as a negative control. Cells were collected 8 hours post treatment and analyzed by RT-Q-PCR as above. Human sera did not itself activate DCs or MØ. (**A, B, C and D**) The data shown are representative of three independent experiments using three blood donors and presented as mean ± sd of triplicates. Statistical significance (paired-sample *t* test) compared to GP-HS is shown as follows: * - p<0.05 and ** - p<0.01; n.s. – not significant.

To address whether shed GP binding to immune cells results in their activation, monocyte-derived DCs and Mø were incubated with samples of culture supernatants containing the soluble EBOV glycoproteins detailed above and expression of TNFα, IL6, IL10 and IL12p40 mRNA was measured by real-time PCR at 4, 8, 12 and 24 h post-treatment. In controls, cells were treated either with culture supernatants from cells expressing GFP (Mock) or LPS (500 pg/ml). Culture supernatants from cells expressing GP-LS were used as an additional negative control. Prior to experiments, all culture supernatants were tested for the absence of endotoxin and showed endotoxin concentrations below 0.25 units/ml and were thus considered to be endotoxin-negative. In a sharp contrast to all negative controls used in this experiment, treatment of DCs and Mø with either shed GP or LPS results in transcriptional activation of a number of analysed genes (Figure 3A and B). Upregulation of TNFα and IL6 occurred as early as 4 h post treatment whereas IL10 and IL12p40 were activated only 8 h and 12 h post treatment, respectively. The increase in mRNA levels appears to be higher in Mø than in DCs and when compared to LPS, cell activation caused by shed GP appears to be more durable. Treatment of the cells with sGP did not reveal any significant effects on cytokine mRNA synthesis. In agreement with experiments concerning shed GP binding, pre-treatment of both DCs and Mø with anti-TLR4 antibody significantly reduced shed GP-induced activation of TNFα mRNA synthesis (Figure 3C). A similar neutralizing effect of anti-TLR4 antibody was observed in the case of LPS treatment, in agreement with previous publications demonstrating that LPS acts on cells via TLR4 [57], [64]. Furthermore, as shown in Figure 3D, pre-treatment of shed GP with human sera containing MBL results in the abrogation of shed GP-dependent TNFα activation. Importantly experiments with dose-dependent inhibition of shed GP activity by MBL-containing sera clearly support the conclusion that the inhibitory effect of sera lectins will be countered by the continuously growing amounts of shed GP in vivo (Figure 3D). A two-fold decrease in MBL amount led to a significant reduction of the MBL inhibitory effect on the activation of both DCs and macrophages by shed GP. No blocking effect was observed after pre-treatment of shed GP with MBL-deficient sera. As expected, both human sera used did not themselves activate DCs or Mø.

### EBOV shed GP induces release of pro- and anti-inflammatory cytokines from DCs and Mø

**Figure 4:**
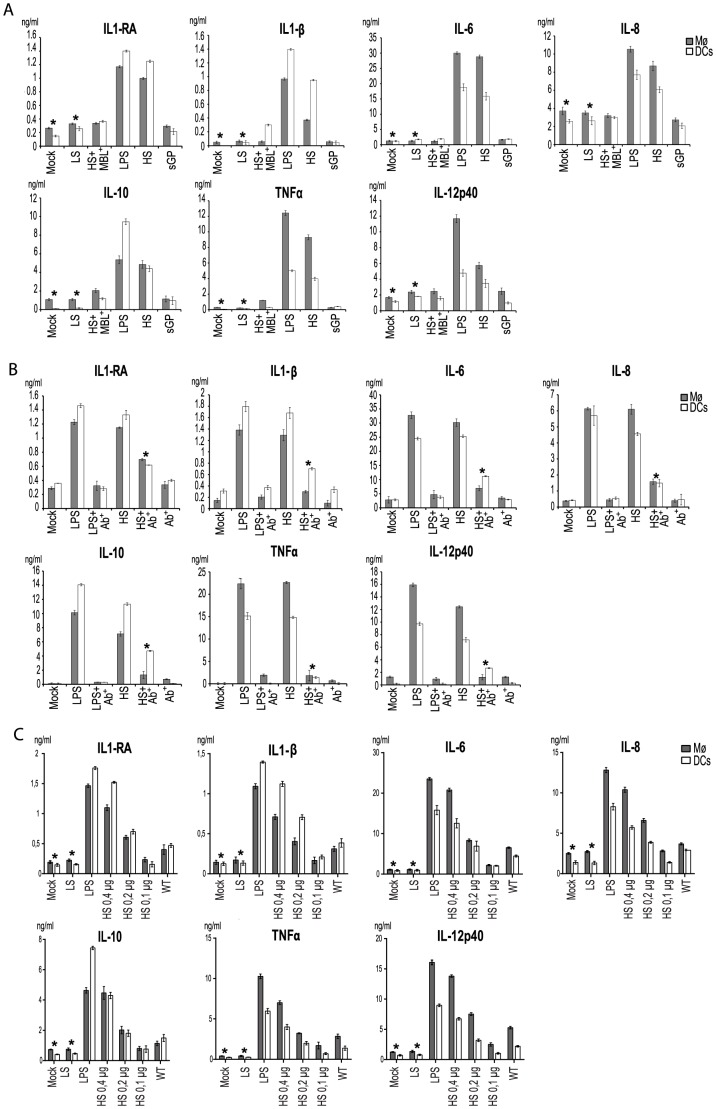
Shed GP induces secretion of cytokines from DCs and MØ. (**A**) 5×10^5^ of DCs (in white) and MØ (in gray) were incubated for 24 h with concentrated culture supernatants as in Figure 2. (**B**) 5×10^5^ of DCs (in white) and MØ (in gray) were incubated for 24 h with concentrated culture supernatants as above and, in addition, the cells were pre-incubated with an anti-TLR4 antibody (Ab^+^) followed by treatment with HS or LPS. (**C**) Shed GP induces secretion of cytokines from DCs and MØ in a dose-dependent manner. 5×10^5^ of DCs (in white) and MØ (in gray) were incubated for 24 h with concentrated culture supernatants, or LPS as above. Decreasing quantities of shed GP (HS) ranging from 0.4 µg to 0.1 µg were tested as indicated. (**A, B and C**) The concentration of each cytokine in duplicate was measured using Multiplex cytometric bead array (Bio-Rad) in Luminex MAGPIX. The data shown are representative of three independent experiments with three blood donors and presented as mean ± sd of triplicates. Statistically significant differences (paired-sample *t* test) compared to GP-HS are shown as follows: * - p<0.05.

Given the transcriptional activation observed above it follows that upon stimulation DCs and Mø would release a range of cytokines into the medium. Accordingly, culture supernatants of DCs and Mø collected 24 h after addition of shed GP (as detailed above) were assayed using a Multiplex cytometric bead array (Bio-Rad) against a panel of cytokines previously shown to be upregulated during EBOV infection [62], [65]. As expected, shed GP induced the secretion of TNFα, IL6, IL10, IL12, IL8, IL1β and IL1RA in both DCs and Mø (Figure 4A). Comparable levels of these cytokines were also observed in the medium when cells were treated with LPS. All negative controls used in this study including samples of medium from GP-LS expressing cells did not show any significant release of the cytokines. Incubation of the cells with the sGP-containing sample also did not result in detectable cytokine secretion. Expectedly, pre-treatment of shed GP-containing samples with MBL resulted in a complete block in cytokine release that correlates well with the absence of activation of cells, as demonstrated in figure 3A and 3B. In accordance with the results presented above, pre-treatment of cells with an anti-TLR4 antibody considerably reduced the release of TNFα, IL6, IL10, IL12, IL8, IL1β and IL1RA (Figure 4B). Again a similar neutralizing effect of anti-TLR4 antibody was observed in the case of LPS treatment. Importantly, shed GP produced in 293T cells cultured with 5% FCS also failed to show activation of immune cells (Figure S4) supporting the importance of using sera-free medium for shed GP production.

In a separate experiment we determined that shed GP activity is dose-dependent. Several dilutions of shed GP from GP-HS expressing cells as well as from wild-type GP-expressing cells were used to treat immune cells and culture supernatants from DCs and Mø collected 24 h after addition of shed GP were assayed (as detailed above) using a Multiplex cytometric bead array (Bio-Rad) against a panel of cytokines. As shown in figure 4C the activity of shed GP released from wt GP- and GP-HS expressing cells is comparable when the concentration of the proteins is adjusted. This also demonstrates that whilst increasing GP shedding efficacy the single amino acid exchange at the carboxy-terminal end of GP-HS does not affect the protein's activity in comparison to the wild-type sequence.

**Figure 5:**
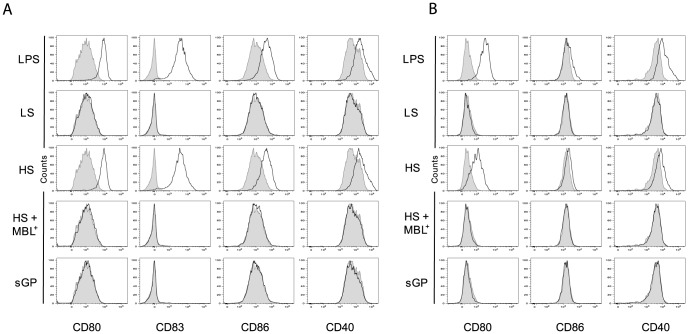
Shed GP induces the phenotypic maturation of DCs and MØ. 5×10^5^ of DCs (**A**) and macrophages (**B**) were incubated with concentrated culture supernatants as above. The cells were harvested at 48 h post-incubation and expression of CD80, CD86, CD40 and CD83 was analyzed by flow cytometry. Filled histograms represent staining with an appropriate isotype-matched control antibody. Data shown are representative of three independent experiments using three blood donors. For quantitative data and statistics see figure S5.

As activation of DCs and Mø is usually associated with an increase in expression of phenotypic activation markers such as the co-stimulatory molecules CD86, CD80, CD83 and CD40, we investigated whether the binding of shed GP or sGP to these immune cells could induce the expression of these surface molecules. As demonstrated in figure 5A and B and figure S5A and B, exposure to shed GP but not to sGP induced the expression of co-stimulatory molecules on both DCs and Mø. Similar expression of surface markers was observed upon treatment of the cells with LPS. Samples of culture medium from GP-LS expressing cells, used here as a negative control, did not reveal any significant effect on immune cells. Pre-treatment of cells with MBL prevents cell activation as demonstrated by the absence of any increase in co-stimulatory molecule expression.

### Glycosylation of shed GP is vital to activate DCs and Mø

**Figure 6:**
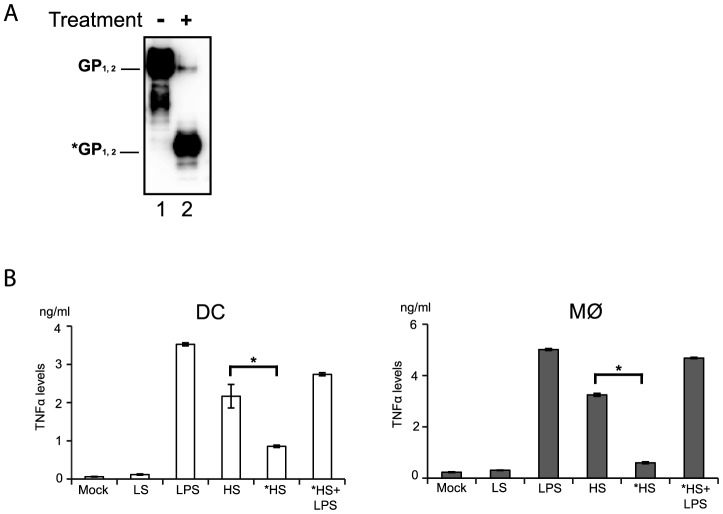
Deglycosylation of shed GP affects activation of DCs and macrophages. (**A**) Concentrated culture supernatants of 293T cells expressing GP-HS were treated either with inactivated deglycosylation enzymes (lane 1) or a mix of deglycosylases (lane 2). Samples were analyzed by Western blot under non-reducing conditions. Deglycosylated EBOV GP is indicated as *GP_1,2Δ_. (**B**) 5×10^5^ DCs (in white) and MØ (in gray) were incubated for 24 h with concentrated culture supernatants of 293T cells expressing GP-HS (HS), GP-LS (LS), and GFP (Mock) after treatment with inactivated deglycosylation enzymes, LPS as a positive control and HS after treatment with a mix of deglycosylation enzymes (*HS). In addition the cells were incubated with *HS following LPS treatment (*HS+LPS). The concentration of TNFα in duplicate was measured using Multiplex cytometric bead array (Bio-Rad) in Luminex MAGPIX. The data shown are representative of three independent experiments with three blood donors and presented as mean ± sd of triplicates. Statistical significance (paired-sample *t* test) compared to GP-HS is shown as follows: * - p<0.05.

Since anti-TLR4 antibodies were capable of reducing shed GP binding and also of decreasing immune cell activation we speculated that GP glycosylation might serve as a pathogen associated molecular pattern (PAMP) recognized by TLR4. Indeed, the glycosylation pattern of EBOV GP is complex and composed of mature and immature N-glycans but also a dense cluster of O-glycosylated serine and threonine residues referred to as mucin-like domain [18], [19]. In order to verify this idea shed GP was treated under non denaturating and non-reducing conditions with a mix of N- and O- glycosidases and then used for incubation with immune cells, as detailed above. As shown in figure 6A the treatment with deglycosylation enzymes reduced the molecular weight of shed GP as expected, in comparison to shed GP treated with the same mix of deglycosidases that were previously inactivated by heating for 30 min at 80°C (Figure 6A, compare lanes 1 and 2). DCs and Mø were incubated with shed GP samples or LPS alone for 12 h, and then culture supernatants were analyzed by ELISA for amounts of secreted TNFα. As an additional control cells were subjected to incubation with LPS following incubation with deglycosylated shed GP. Strikingly, as shown in Figure 6B, deglycosylation of shed GP considerably decreased the release of TNFα in both DCs and Mø, confirming the importance of the glycosylation pattern of shed GP for its activity. Cells incubated with deglycosylated shed GP remained viable and capable of responding to LPS stimuli ruling out a possible negative effect of deglycosydases on cells.

Taken together these results suggest that the particular glycosylation pattern of shed GP is responsible for its functions as an inducer of immune cell activation and that this function is likely mediated via shed GP binding to TLR4 present on DCs and Mø acting as a pathogen pattern recognition receptor. This would appear consistent with the hypothesis that shed GP is acting in a manner somewhat similar to that seen for LPS in its ability to cause systemic inflammation.

### Modulation of endothelial permeability by EBOV shed GP

**Figure 7:**
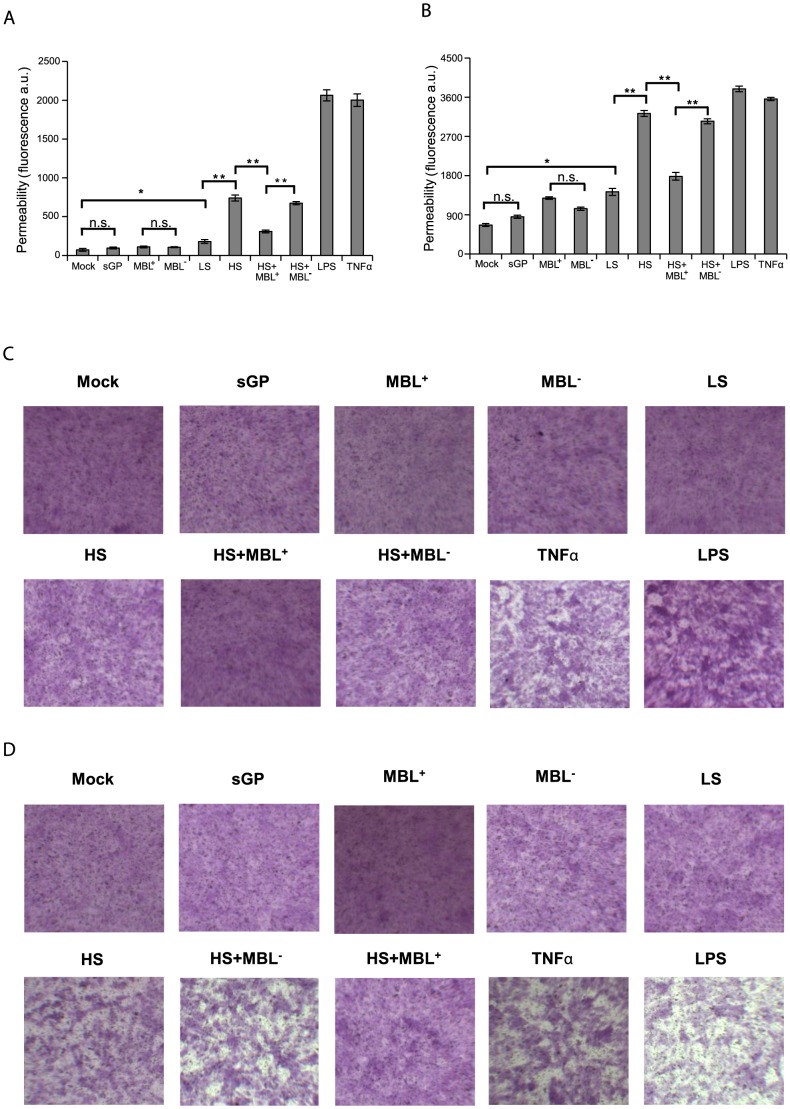
EBOV Shed GP increases cell permeability of HUVEC in vitro. HUVEC cells were seeded onto the culture inserts of permeability assay chambers and incubated for 22 h with concentrated culture supernatants from 293T cells expressing GFP (Mock), sGP, GP-LS (LS), GP-HS (HS) and LPS (10 ng/ml). Cells were also incubated with human sera containing MBL (MBL^+^), MBL-deficient sera (MBL^−^), a mixture of HS/MBL-containing sera (HS+MBL^+^) and HS/MBL-deficient sera (HS+MBL^−^). As a permeability control the cells were treated with TNFα (20 ng/ml) (**A and C**). In parallel, HUVEC cells were also incubated for 22 h with culture supernatants from macrophages previously stimulated with samples of concentrated culture supernatants and controls as above. As a permeability control the cells were treated with TNFα (100 ng/ml) (**B and D**). In all cases following incubation, FITC-Dextran was added to each insert and FITC-Dextran leaking out into the bottom chamber was assayed using a fluorometer and presented as absorbance fluorescent units. Bars represent ± SD of triplicates. The data are representative of three independent experiments. * indicates statistically significant differences (paired-sample *t* test, * - p<0.05 and ** - p<0.01) or n.s. - statistically not significant (**A and B**). The integrity of HUVEC monolayers assayed in A and B was also observed with a MacroFluo microscope after fixation and coloration (**C and D**).

Vascular dysregulation and instability are thought to be crucial symptoms during EBOV infection [24], [66]. While endothelial cells are susceptible EBOV targets in vitro [35], [67], they are considered to be rather late virus targets in vivo [68], [69] suggesting an indirect effect of EBOV infection on this cells. A dysregulated inflammatory host response to EBOV infection is believed to facilitate increased permeability of endothelial barriers that would eventually cause haemorrhaging [67], [70]. In this regard proinflammatory cytokines, in particular TNFα, released from immune cells activated by shed GP could affect endothelial barrier integrity [68], [70]. Furthermore, as we demonstrated that shed GP is capable of activating immune cells for release of different cytokines, we also sought to determine if shed GP could directly affect endothelial cell permeability. To answer these questions, an *in vitro* monolayer cell permeability assay was performed using HUVECs. Firstly, HUVEC monolayers grown on semi-permeable membranes in cell culture plate inserts were treated for 22 h with samples containing soluble EBOV glycoproteins. Following treatment FITC-dextran was added to the apical part of the insert and the permeability of the monolayer (Figure 7A) was assessed by measuring fluorescence in the basal compartment. As controls, cells were treated with samples of culture medium from GFP- (Mock), sGP- or GP-LS-expressing cells and also with LPS (as above), MBL-containing sera, MBL-deficient sera and TNFα (10 ng/ml). The integrity of HUVEC monolayers was verified with a fluorometer both before and after 22 h of incubation with supernatants containing soluble glycoproteins. HUVEC monolayers were also photographed under a light microscope (Figure 7C). As seen in Figure 7A and 7C, samples containing GP-HS, LPS and TNFα significantly increased the permeability of the HUVEC monolayer when compared with negative controls and sGP. As expected, culture supernatants containing GP-HS in the presence of MBL-containing sera were significantly less capable of inducing an increase in permeability of the HUVEC monolayer compared to MBL-deficient sera. Treatment with human sera alone did not show any significant effect on permeability either with or deficient for MBL. Secondly, we also assayed whether the levels of soluble modulators released from macrophages treated with samples of shed GP and all controls (as above) were sufficient to contribute to the decreased barrier function of the endothelium. As seen in figure 7B and D, the supernatants from shed GP-treated macrophages significantly increased the permeability of the HUVEC monolayer when compared to samples of culture medium from GFP- (Mock) or sGP-expressing cells, in a manner comparable with that seen with LPS-treated macrophages or when TNFα was used as a control. Somewhat surprisingly given the absence of detectable activation in previous assays, a moderate increase in permeability was observed when culture medium from GP-LS-expressing cells was used, probably explained either by residual amounts of shed GP present in GP-LS samples used to treat macrophages or by the presence of another undetermined factor released during cell treatment with medium upon GP expression. Culture supernatants from macrophages treated with shed GP in the presence of MBL containing sera were significantly less capable of inducing an increase in permeability of the HUVEC monolayer compared to MBL-deficient sera. Treatment with human sera alone did not show any significant effect on permeability either with or deficient for MBL. The ability of shed GP to affect endothelial cells function both directly and indirectly highlights the interplay between shed GP, systemic cytokine release and increased vascular permeability.

Taken together the data obtained support a role for EBOV shed GP in the creation of excessive and dysregulated host inflammatory responses and an increased vascular permeability. Based on the results presented here we postulate that once released from the surface of infected cells shed GP could be responsible for the induction and release from non-infected macrophages and DCs of a battery of pro- and anti-inflammatory cytokines. This is especially interesting as elevated levels of pro- and anti-inflammatory cytokines have been shown to be present in fatal EBOV infections in man [62], [71]. Importantly, a clear link has been demonstrated between clinical status and inflammatory responses in that fatal outcome of infection is often associated with aberrant innate immune responses [71], [72].

This study provides a potential explanation as to the cause of these disregulated host responses, since it has been demonstrated that neither the whole virus nor viral replication in immune cells could explain an excessive activation of immune cells for release of these cytokines [30], [62], [71], [73]. We now show that cultured primary DCs and macrophages are activated upon treatment with EBOV shed GP, resulting in release of several mediators including pro- and anti-inflammatory cytokines such as TNFα, IL6, IL10, IL12, IL8, IL1β and IL1RA independently of virus replication in these cells.

The apparent similarity in the response of immune cells to shed GP and LPS highlights a likely common triggering of TLR4 and shared downstream pathway for activation and cytokine release. Intriguingly, a common feature of both LPS as a bacterial component and EBOV infection is their ability to trigger a similar physiological syndrome typified by elevated cytokine release, which for EBOV infection closely resembles the septic shock seen with bacterial infection. Indeed, when presented in VLPs EBOV GP has been shown to interact with TLR4, leading to the induction of proinflammatory cytokines as well as SOCS1 in both a human monocytic cell line (THP-1) and 293T cells expressing a functional TLR4/MD2 receptor [52]. Furthermore, a recent study by Lubaki et al. [30] provided clear evidence that an unrelated viral vector carrying EBOV GP could effectively cause maturation of dendritic cells. However, during EBOV replication in dendritic cells interferon antagonism mediated by virus-encoded proteins VP35 and VP24 strongly reduces both the expression of maturation markers and secretion of cytokines and chemokines from these cells. On the contrary, EBOV shed GP as a soluble mediator is able to activate non-infected immune cells, making it distinct from a number of other viruses whose surface glycoproteins have been shown to act as ligands for TLR4 recognition but that either do not shed their surface glycoproteins into the extracellular environment or do not spread systemically and thus only cause local inflammatory disorders [74]–[78].

**Figure 8:**
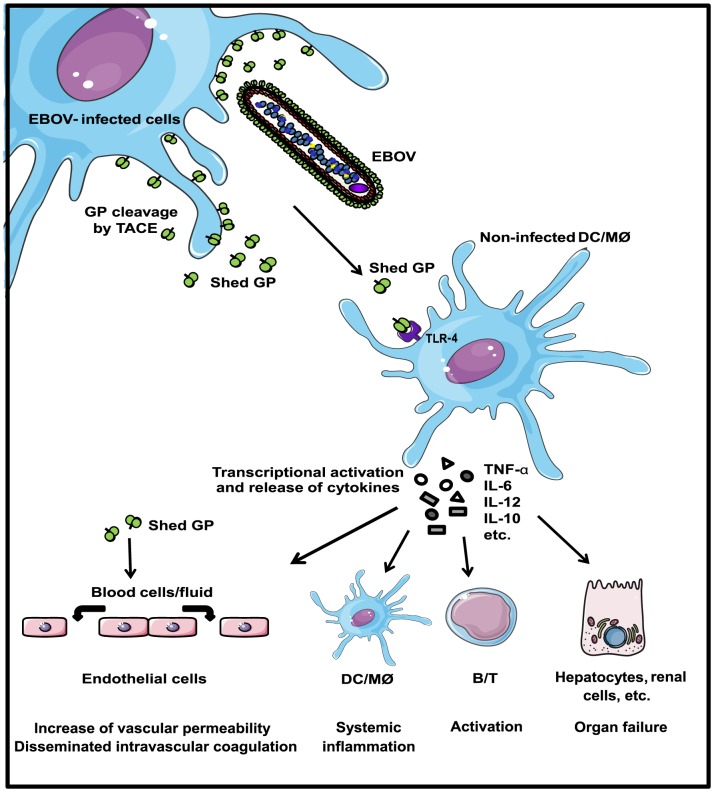
Role of shed GP during EBOV infection. In our model, EBOV-infected cells release both virions and soluble shed GP. Shed GP binds DCs and macrophages using TLR4 and activates these cells for release of pro- and anti-inflammatory cytokines. The soluble nature of shed GP enables triggering of additional and remote targets such as non-infected APCs. Release of cytokines from these cells will further amplify inflammation by recruiting and activating other APCs, lymphocytes, endothelial cells, and other cell types including hepatocytes. Overall the release of shed GP is likely to contribute to increased vascular permeability, disseminated intravascular coagulation, disregulated inflammation and impairment of cell function and organ failure.

In conclusion, in the present study we have identified the primary targets of EBOV soluble glycoprotein shed GP. In our model, EBOV infected cells release both virions and shed GP into extracellular environment. Shed GP, likely via interaction with TLR4 on the surface of macrophages and DCs induces activation of these cells and the release of pro- and anti-inflammatory cytokines. The soluble nature of both shed GP and cytokines enables them to trigger new, remote targets such as non-infected APCs, which will then amplify inflammation by recruiting and/or activating other cell types including lymphocytes, endothelial cells and other cell types including hepatocytes, leading to the impairment of cell functions and organ failure (Figure 8). Our results strongly suggest that the target cell range for EBOV infection is considerably broader than previously imagined. In effect, activation of non-infected immune cells by shed GP could play an important role in the establishment of systemic inflammation during infection. Furthermore the data obtained with shed GP provide a potential link between systemic inflammation and increased endothelial permeability that together contribute to the disseminated intravascular coagulation syndrome seen with lethal EBOV infection. Overall, our data contribute to a better understanding of the way EBOV might provoke the excessive cytokine storm that appears to be detrimental to survival of infection and provide new insights with which to develop therapeutic strategies to combat this newly defined role for shed GP in high viral pathogenicity. In this regard, it is intriguing to speculate that treatment with anti-TLR4 antibodies could be used to reduce the inflammatory reaction caused by shed GP in a similar way than has been demonstrated for the dramatically successful treatment of septic shock in mice after lethal LPS challenge [79]. Similarly, it is conceivable that neutralizing antibodies targeting shed GP could also help to alleviate the systemic shock-like syndrome seen with EBOV infection.

## Materials and Methods

### Virus

The work with infectious EBOV belonging to *Filoviridae* family, *ebolavirus genus, species Zaire Ebolavirus* was performed at INSERM “Laboratoire Jean Mérieux” within biosafety level 4 containment at Lyon, France. The virus was propagated on Vero E6 cells. Culture supernatants were collected, clarified, frozen and kept at -80°C before virus titration by TCID_50_ [80] The virus was generated using a reverse genetics system previously described in [81]. Amino acid substitutions M71I, L147P, T187I were introduced into the VP24 gene to increase the pathogenicity in recombinant EBOVs for guinea pigs [37], [82]. Mutations introduced into the virus were confirmed by sequencing of complete genomic RNA isolated from virus stocks.

### Cell lines and plasmids

293T cells were cultured in Dulbecco's modified Eagle's medium (DMEM, PAA laboratories) supplemented with 10% fetal calf serum (PerbioHyclone). Human umbilical vein endothelial cells (HUVECs) (Lonza) were maintained at 37°C with 5% CO2 in EGM-2 Media (Lonza) enriched with EGM-2 Single Quots containing FBS, hydrocortisone, hFGF-B, VEGF, R3-IGF-1, ascorbic acid, hEGF, GA-1000 and heparin. Cells were cultured for 3–4 days with EGM-2 medium and were passaged for no more than 5 generations for the experiments described.

We have previously demonstrated that the substitutions L635V and D637V dramatically alter the efficacy of EBOV GP shedding in a transient expression system [21]. In this study to increase the efficiency of GP shedding in a transient expression system we introduced the substitutions D637A and Q638V using site-directed mutagenesis of a plasmid encoding wild-type GP. The resulting plasmid was designated phCMVGP-HS. Plasmid expressing GP with a substitution L635V (phCMVGP-LS) was described earlier [21]. Plasmid expressing transmembrane-anchor truncated mutant GP_1,2Δ_ was generated by introducing a stop codon at position 651 using site-directed mutagenesis of a plasmid encoding wild-type GP.

### Transient expression of EBOV GP

293T cells were grown for 24 h to a confluence of ∼60%. Transfection of cells with recombinant plasmids was performed using TurboFect reagent (Thermo). During GP expression the cells were kept in VP-SFM medium (Gibco) containing no serum. Culture supernatants were harvested 36 h posttransfection, clarified by centrifugation at 1000 rpm for 10 min at 4°C and then by centrifugation at 28,000 rpm at 4°C for 2 h using a SW28 rotor with a Beckman Optima L-70K ultracentrifuge. The clarified supernatants were concentrated 10 fold using Concentrator tubes (Pierce) with a molecular weight cut off of 20000. All samples were aliquoted and stored at −80°C.

### Culture of primary human monocyte-derived-DCs and macrophages

Briefly, peripheral blood mononuclear cells (PBMCs) were isolated from the blood of healthy donors by centrifugation onto a lymphocyte separation medium (Ficoll) cushion and then were enriched by centrifugation of PBMCs through a Percoll density gradient. The recovered monocyte fraction was depleted of cell contaminants by positive selection (Miltenyi Biotec), ensuring purification rates of ≥95%. Immature DCs and monocyte-derived macrophages were obtained upon incubation of cells in complete RPMI-1640 (Life Technologies) supplemented with 200 mM L-glutamine (Life Technologies), 10 mM HEPES buffer (Life Technologies), 10% de-complemented FCS and 8 µg/ml of Gentamicine (Life Technologies) for 5 days either with 40 ng/ml of granulocyte- macrophage colony-stimulating factor (GM-CSF) or 50 ng/ml of interleukin-4 (IL-4) and 40 ng/ml of macrophage colony-stimulating factor (M-CSF), respectively.

### Endotoxin test

The Limulus amebocyte lysate test (LAL Chromogenic Endotoxin Quantitation Kit, Pierce) was used to determine endotoxin levels in culture supernatants used in this study. The values were less or equal to those of VP-SFM medium (<0.25 endotoxin units per ml).

### Sedimentation analysis

The oligomeric structures of EBOV shed GP and transmembrane-anchor truncated mutant GP_1,2Δ_ were compared using a sedimentation assay. 293T cells were transfected with plasmids phCMVGP-HS or phCMVGP_1,2Δ_ and culture medium was collected 36 h post-transfection. Medium was clarified by low-speed centrifugation and subjected to ultracentrifugation through a linear 5–25% (w/w) sucrose gradient prepared on co-IP buffer (1% NP40, 0.4% deoxycholate, 5 mM EDTA, 100 mM NaCl, 20 mM Tris–HCl, pH 7.5, 25 mM iodacetamide) on Beckman Optima L-70K ultracentrifuge using a SW60 rotor for 21 h at 40 000 rpm, 10°C. Fractions were collected from the bottom to the top, proteins separated by SDS–PAGE and analyzed by Western blot using anti-GP antibodies.

### Binding assay

5×10^5^ monocyte-derived DCs or macrophages per well were placed in conic-bottom 96-well plates and plates were centrifuged at 2000 rpm for 2 min. The culture medium was removed and replaced by medium containing EBOV GPs (in VP-SFM medium, Gibco). Cells were incubated for 1 hour at 4°C, washed twice with ice-cold phosphate-buffered saline (PBS) and then incubated in FACS buffer (PBS containing 2% FBS, 2% normal rat serum, 2% normal hamster serum, 2% normal mouse serum and 2% human FcR blocking solution, Milteny Biotec) at 4°C for 30 min. Cells were then incubated with mouse anti-GP1 antibody for 1 hour at 4°C, washed twice with PBS and incubated with anti-mouse IgG1 coupled to Alexa 488 for DCs and Mø or to Allophycocyanin (APC) for lymphocytes for 30 min at 4°C. All mAbs were purchased from BD Pharmingen except those indicated otherwise. After incubation with antibodies, cells were washed and fixed with 1% PFA solution. Cells were analyzed using a Becton Dickinson LSRII flow cytometer (BD) and FlowJo software. In some experiments samples of GP were incubated with human sera containing MBL (150 ng/ml) or MBL- deficient sera (Statens Serum Institute, Denmark) for 1 hour at 4°C prior to addition to immune cells. In TLR4-related experiments, cells were incubated for 10 min at room temperature with 30 µg/ml of anti-TLR4 antibodies (HTA125) or unspecific isotype antibody prior to addition of EBOV GPs. The statistical significance of the differences in median fluorescence intensity (MFI) values for each donor were evaluated by paired-sample *t* test and GraphPad Prism 6 software (GraphPad Software, La Jolla California, USA).

### Treatment of cells with EBOV GPs

5×10^5^ of monocyte-derived DCs or macrophages per well were placed in 48-well plates and after 5 days differentiation, media was replaced with 600 µl of fresh RPMI 1640 containing 200 mM L-glutamine, 10 mM HEPES buffer, 5 mM CaCl2 and 400 µl of culture supernatants containing soluble EBOV glycoproteins (in VP-SFM medium). Cells were incubated for 4, 8, 12 and 24 h at 37°C and culture supernatants were collected, clarified from cell debris by centrifugation (1600 rpm at 4°C for 10 min), aliquoted, and stored at −80°C until analysis. The cells were used for real time RT-PCR analysis. Experiments using either anti-TLR4 antibodies or sera containing MBL were performed as described above in Binding assay.

Unless otherwise stated shed GP was used at a final concentration of 0.4 µg/ml and sGP at 7.2 µg/ml. In deglycosylation experiments shed GP was used at a concentration of 0.2 µg/ml.

### Real time RT-PCR quantitative analysis

RNAs from monocyte-derived DCs and macrophages treated with EBOV GPs were isolated using Total RNA Isolation kit (Machery Nagel). cDNAs were generated using iScript cDNA Synthesis kit (Bio-Rad), followed by real-time PCRs using Sybr-Green-Master-Mix (Roche) in triplicate following manufacturer's recommendations. Validated RT-PCR primers specific for human TNFα (5′-CCTGCCCCAATCCCTTTATT and 5′-CCCTAAGCCCCCAATTCTCT), IL-6 (5′-TGCAATAACCACCCCTGACC and 5′-TGCGCAGAATGAGATGAGTTG), IL10 (5′-GAGGCTACGGCGCTGTCAT and 5′-CCACGGCCTTGCTCTTGTT), IL12p40 (5′-CCAGAGCAGTGAGGTCTTAGGC and 5′-TGTGAAGCAGCAGGAGCG) and GAPDH (5′-CCATGTTCGTCATGGGTGTG and 5′-GGTGCTAAGCAGTTGGTGGTG) were used to quantify mRNA levels. The real-time PCR analysis was performed on Stepone Plus apparatus using Roche software. Relative quantification was made by normalization to GAPDH mRNA levels.

### Cytokine release

Culture supernatants from monocyte-derived DCs or macrophages treated with soluble EBOV GPs for 24 hours were assayed for presence of TNF-α, IL-6, IL-8, IL12p40, IL1β, IL10 and IL1-RA using Multiplex cytometric bead array (Bio-Rad) in Luminex MAGPIX. The assay was performed according to the manufacturer's instructions.

### Expression of co-stimulatory molecules

Both monocyte-derived DCs and macrophages treated with soluble EBOV GPs for 48 hours were washed in cold PBS supplemented with 2% fetal bovine serum and pre-blocked at 4°C for 30 min in FACS buffer. Cell-surface staining was performed using the following antibodies: FITC-conjugated anti-CD80, PE-conjugated anti-CD40 (Beckman Coulter) and APC-conjugated anti-CD83, Peridinin Chlorophyll Protein Complex Vio 700 (PerCP-Vio700)-conjugated anti-CD86 (Miltenyi Biotec). After incubation with antibodies for 1 hour at 4°C, cells were washed with PBS, fixed with 1% PFA solution and analyzed using a Becton Dickinson LSRII flow cytometer (BD) and FlowJo software. A minimum of 10000 events were collected and analyzed for each sample. The statistical significance of the differences in median fluorescence intensity (MFI) values for each donor were evaluated by paired-sample *t* test and GraphPad Prism 6 software (GraphPad Software, La Jolla California, USA).

### Deglycosylation assay

For GP deglycosylation, supernatants containing EBOV GPs were treated using EDEGLY kit (Sigma) for 48 h according to the manufacturer's instructions. Shed GP mixed with deglycosylation enzymes pretreated at 80°C for 30 min were used as a control. Monocyte-derived DCs and macrophages were treated with deglycosylated shed GP samples for 24 hours and culture supernatants were assayed for the presence of TNFα using a Multiplex cytometric bead array (Bio-Rad) in Luminex MAGPIX.

### HUVEC permeabilization assay

HUVEC were grown in collagen-coated inserts of permeability assay chambers (Millipore) for 72 h until confluence. The media was replaced with 600 µl of fresh EBM (endothelial cell basal medium without phenol red, Lonza) and 400 µl of culture supernatants containing soluble EBOV glycoproteins (VP-SFM medium, Gibco) or 400 µl of culture supernatants from Mø previously stimulated with soluble EBOV glycoproteins for 24 h. After 22 h of incubation, FITC-Dextran was added to each insert and FITC-Dextran leaking out into the bottom chamber was measured. Monolayer permeability was assessed on the basis of both fluorescence intensities measured using a Tecan fluorometer and the integrity of the HUVEC monolayer using a MacroFluo microscope.

### Animal experiments

Guinea pigs, strain Hartley (3 week-old females), were infected intraperitoneally with 500 TCID_50_ of recombinant guinea-pig adapted EBOV as previously described [82]. Mock-infected controls were inoculated with DMEM. Animals were monitored for clinical manifestations and were euthanized when they reached an ethical end*-*point. Retro-orbital sinus sampling of blood was performed after local anaesthesia at day 3, 6 and 9 and prior to euthanasia. The sera were purified using VenoSafe PET tubes (VenoSafe).

All animals were handled in strict accordance with good practices as defined by the French national charter on the ethics of animal experimentation. Animal work was approved by the regional ethical committee (CREEA), and experiments were performed in the INSERM Jean Mérieux BSL-4 laboratory in Lyon, France.

## Supporting Information

Figure S1 (**A and B**) Quantification of shed GP (A) and sGP (B) produced in GP-HS- and sGP-expressing 293T cells. Serial dilutions of recombinant EBOV GP as indicated, produced in insect cells (recGP, IBT Bioservices) were used to estimate amounts of HS and sGP in concentrated culture supernatants. Samples of GP and sGP were analyzed by Western blot using anti-GP1 antibodies and ImageQuantTL software (GE Lifesciences). As illustrated the standard curve was linear with an R2 value >0.99 over the range used for quantification (open diamonds). Note: the molecular mass of shed GP is ∼160 kDa, sGP ∼55 kDa, in comparison to recGP of ∼110 kDa. Shed GP sample (closed square) corresponds to ∼11.3 ng of recGP and thus contains ∼1 µg/ml in the concentrated supernatants. sGP sample (closed square) corresponds to ∼44 ng of recGP and thus contains ∼18 µg/ml of sGP.
(EPS)

Figure S2 (**A**) Schematic representation of EBOV surface GP, shed GP and a truncated GP mutant (GP_ΔTM_) containing a stop codon immediate upstream of the transmembrane anchor. (**B**) Sedimentation analysis. Samples of shed GP and GP_ΔTM_ were subjected to centrifugation through 5–25% sucrose gradients followed by analysis of gradient fractions using Western blot and anti-GP antibodies. Fractions 1–2 correspond to GP trimers and 5–7 to GP monomers. The orientation of the gradient is shown.
(EPS)

Figure S3 Quantitative data and statistical analysis of data presented in Figure 2. EBOV shed GP binding to DCs and macrophages. (**A**) Human monocyte-derived dendritic cells (DCs), monocyte-derived macrophages (MØ), and PBLs (shown B lymphocytes, B) were incubated with shed GP as well as with shed GP in the presence of MBL-containing sera (150 ng/ml, HS+MBL^+^), as described in Figure 2. Bound proteins were detected by subsequent incubation with mouse anti-GP1 antibodies and anti-mouse Alexa 488 coupled antibodies (DCs and MØ) and anti-mouse APC (B lymphocytes). Fraction of B lymphocytes was stained using CD20-FITC antibodies (Beckman Coulter). (**B**) DCs and MØ were either incubated with supernatants containing GP-HS (as above) or were pre-treated with anti-TLR4 antibody (Ab^+^) or isotypic control antibodies (Ab^−^) prior to shed GP treatment. (**C**) DCs and MØ were incubated with serum containing 150 ng/ml of MBL-containing sera (MBL^+^), MBL-deficient sera (MBL^−^) or culture media alone before washing and incubation with shed GP (as above). (**A, B and C**) Shed GP binding to cells was analyzed by flow cytometry and shown as raw MFI data for at least three independent blood donors. Statistically significant differences compared to HS are shown as follows: * - p<0.05 and ** - p<0.01; n.s. – not significant.
(EPS)

Figure S4 EBOV shed GP containing sera does not activate DCs and MØ. Human monocyte-derived dendritic cells (DCs) and monocyte-derived macrophages (MØ) were incubated with either shed GP as above (HS+0%) or with shed GP in the presence of 5% bovine sera (HS+5%). As control, the cells were incubated with LPS or concentrated culture supernatants from GFP expressing cells (Mock). Statistically significant differences (paired-sample t test) compared to HS+0% are shown as follows: * - p<0.05.
(EPS)

Figure S5 Quantitative data and statistical analysis of data presented in Figure 5. Shed GP induces the phenotypic maturation of DCs and MØ. 5×10^5^ of DCs (**A**) and macrophages (**B**) were incubated with concentrated culture supernatants. The cells were harvested at 48 h post-incubation and expression of CD80, CD86, CD40 and CD83 was analyzed by flow cytometry. Shed GP binding to cells was analyzed by flow cytometry and shown as raw MFI data for at least three independent blood donors. Statistically significant differences compared to HS are shown as follows: * - p<0.05 and ** - p<0.01; *** - p<0.001.
(EPS)
